# Supplementary material for: Superior robustness of anomalous non-reciprocal topological edge states
Source: Nature. 2021 Oct 13;598(7880):293–7. doi: 10.1038/s41586-021-03868-7 (PMC8514337; doi:10.1038/s41586-021-03868-7)
Supplement: Supplementary file 1 — This file contains six Supplementary Discussion sections I–VI, Supplementary Figures 1–11 and Supplementary References. The six Supplementary Discussion Sections include: I. Theoretical modelling; II. Simulation method of arbitrary finite non-reciprocal networks; III. Examples of disorder realisations for phase-link delays and scattering matrices; IV. Discussion of the measured amplitudes of the scattering parameters in Extended Data Fig. 4; V. Mapping of the honeycomb lattice to an oriented graph and VI. Robustness comparisons with other Chern phases. [file 41586_2021_3868_MOESM1_ESM.pdf]

nature portfolio

https://doi.org/10.1038/s41586-021-03868-7

Supplementary information

Superior robustness of anomalous non-reciprocal topological edge states

In the format provided by the authors and unedited

Supplementary Information for

Superior robustness of anomalous nonreciprocal topological edge states

Zhe Zhang[1], Pierre Delplace[2], and Romain Fleury[1]*

[1]*Laboratory of Wave Engineering, School of Electrical Engineering, EPFL, Station 11, 1015 Lausanne, Switzerland*

[2]*University of Lyon, ENS Lyon, University Claude Bernard, CNRS, Laboratoire de Physique, F-69342 Lyon, France*

**To whom correspondence should be addressed. Email: romain.fleury@epfl.ch*

This supplementary information contains the following sections:

I. Theoretical modelling

II. Simulation method of arbitrary finite nonreciprocal networks

III. Examples of disorder realisations for phase-link delays and scattering matrices

IV. Discussion of the measured amplitudes of the scattering parameters in Extended Data Fig. 4

V. Mapping of the honeycomb lattice to an oriented graph

VI. Robustness comparisons with other Chern phases

VII. Supplementary references

I. Theoretical modelling

We first show how to parameterize a 3-port nonreciprocal element and obtain its scattering-wave model. The 3-port nonreciprocal elements, shown in Fig. 1a and b of the main text, are modelled as lossless 3-port scatterers with C3 symmetry, whose non-reciprocity results from Zeeman-like splitting coming from an external time-odd bias[1–3]. Using temporal coupled mode theory[2], their scattering is represented by an asymmetrical scattering matrix S_0, given by

$$S_0 = -I + \kappa^2 \begin{bmatrix} \sqrt{\gamma_+} & \sqrt{\gamma_-} \\ e^{i\theta}\sqrt{\gamma_+} & e^{-i\theta}\sqrt{\gamma_-} \\ e^{-i\theta}\sqrt{\gamma_+} & e^{i\theta}\sqrt{\gamma_-} \end{bmatrix} \begin{bmatrix} \dfrac{i}{(\omega-\omega_+ + i\gamma_+)} & 0 \\ 0 & \dfrac{i}{(\omega-\omega_- + i\gamma_-)} \end{bmatrix} \begin{bmatrix} \sqrt{\gamma_+} & e^{-i\theta}\sqrt{\gamma_+} & e^{i\theta}\sqrt{\gamma_+} \\ \sqrt{\gamma_-} & e^{i\theta}\sqrt{\gamma_-} & e^{-i\theta}\sqrt{\gamma_-} \end{bmatrix}, \quad \text{(S1)}$$

where I is a 3 by 3 identity matrix, $\kappa = \sqrt{2/3}$, and θ=2π/3, while ω_+ and ω_- represent the eigenvalues of the right-handed and left-handed eigenmodes of the cavity, respectively. Zeeman-like splitting assumes that these two eigenvalues originate from a linear lifting of the two degenerate modes of the cavity by an external magnetic bias, originally at resonant frequency ω_0. γ_+ and γ_- are the inverse of their corresponding decay times to the three ports connected to the outer links, namely waveguides. Without loss, the scattering matrix S_0 is unitary. Due to C_3 symmetry, we obtain the equality $\gamma_+ = \gamma_- = \gamma$. In the above expression, despite being an important parameter for the cavity, γ just introduces a scaling factor to all the frequency parameters (ω, ω_+ and ω_-). In order to show a general parameterization, we transform ω_+ and ω_- into two angle variables ξ and η, by standard normalizations and arctangent transformations:

$$\begin{cases} \xi = \text{atan}\,(\dfrac{\omega-\omega_+}{\gamma}) \\ \eta = \text{atan}\,(\dfrac{\omega-\omega_-}{\gamma}) \end{cases}. \quad \text{(S2)}$$

ξ and η are defined in (-π/2, π/2) with a periodicity of π, and characterize the deviation of the angular frequency ω from the right- and left-handed eigenvalues ω_+ and ω_-, respectively. Specifically, the condition $\xi = \eta$ corresponds to the reciprocal case, with $\omega_+ = \omega_-$, while $\xi = -\eta$ represents the operation at the resonant frequency $\omega = \omega_0$, with the largest non-reciprocity[2]. With the parameters (ξ, η), the scattering matrix in Eq. (S1) is rewritten as:

$$S_0 = -I + \frac{2}{3}\begin{bmatrix} 1 & 1 \\ e^{i\frac{2\pi}{3}} & e^{-i\frac{2\pi}{3}} \\ e^{-i\frac{2\pi}{3}} & e^{i\frac{2\pi}{3}} \end{bmatrix}\begin{bmatrix} \cos\xi \cdot e^{i\xi} & 0 \\ 0 & \cos\eta \cdot e^{i\eta} \end{bmatrix}\begin{bmatrix} 1 & e^{-i\frac{2\pi}{3}} & e^{i\frac{2\pi}{3}} \\ 1 & e^{i\frac{2\pi}{3}} & e^{-i\frac{2\pi}{3}} \end{bmatrix}. \qquad \text{(S3)}$$

As shown in Eq. (S3), the individual reflection coefficient $|R|$ of the nonreciprocal scatterers is a function of ξ and η, expressed as $|R| = \left|-1 + \frac{2}{3}\cos\xi\, e^{i\xi} + \frac{2}{3}\cos\eta\, e^{i\eta}\right|$.

Having established the general scattering-wave model of C3 symmetric unitary 3-port scatterers, we now derive the eigenequation Eq. (1) in the main text, which determines the bulk band structures. We give the detailed schematic of the unit cell of the periodic nonreciprocal network and signal labelling convention in Extended Data Fig. 1. Here, S_A and S_B are the scattering matrices of the two nonreciprocal elements A and B, respectively, governed by the parameterization Eq. (S3). In the following derivations, elements A and B are the same, while the total phase delay between two nonreciprocal elements is φ. For a full description, in a unit cell of a honeycomb lattice with index n, the scattering waves are labelled and arranged into three vectors: $|a_n>$, $|b_n>$, and $|c_n>$, which all contain six complex wave amplitudes and represent scattering waves amplitudes propagating out, in and between the nonreciprocal elements, respectively. Based on the scattering matrices of nonreciprocal element A and B, we relate $|a_n>$ with $|c_n>$ by a unitary matrix $S_{elements}$, expressed as

$$S_{elements}\left|\boldsymbol{c}_n\right\rangle \equiv P_0\begin{bmatrix} & S_A \\ S_B & \end{bmatrix}\left|\boldsymbol{c}_n\right\rangle = \left|\boldsymbol{a}_n\right\rangle, \tag{S4}$$

in which $P_0 = U_{12}U_{13}U_{45}U_{56}\boldsymbol{I}_6$, $\boldsymbol{I}_6$ is a 6 by 6 identity matrix, and U_{ij} is a special permutation matrix which leads the interchange between i th row and j th rows of a matrix A when A is pre-multiplied by U_{ij}. Therefore, P_0 is also a permutation matrix, hence unitary. Here, $S_{elements}$ is a function of (ξ, η), but not of the lattice index n, due to periodicity. With Bloch theory, Eq. (S4) can be written in momentum space as $S_{elements}|c(\boldsymbol{k})>= |a(\boldsymbol{k})>$.

To form the Bloch eigenproblem, we use the phase delay relation induced by the links between the nonreciprocal elements, and form a relation between $|a(\boldsymbol{k})>$ and $|c(\boldsymbol{k})>$,

$$\left|\boldsymbol{a}(\boldsymbol{k})\right\rangle = e^{-i\varphi}\Lambda(\boldsymbol{k})\left|\boldsymbol{c}(\boldsymbol{k})\right\rangle, \tag{S5}$$

where $\Lambda(\boldsymbol{k}) = diag(e^{i\boldsymbol{k}\cdot\boldsymbol{\alpha}_2}, e^{i\boldsymbol{k}\cdot\boldsymbol{\alpha}_1}, 1, 1, e^{-i\boldsymbol{k}\cdot\boldsymbol{\alpha}_2}, e^{-i\boldsymbol{k}\cdot\boldsymbol{\alpha}_1})$ is also unitary. Substituting Eq. (S5) into Eq. (S4), we finally arrive the eigenequation Eq. (1) of the main text

$$S(\boldsymbol{k})\left|\boldsymbol{c}(\boldsymbol{k})\right\rangle = e^{-i\varphi}\left|\boldsymbol{c}(\boldsymbol{k})\right\rangle, \tag{S6}$$

where $S(\boldsymbol{k}) = \Lambda^{-1}(\boldsymbol{k})\, S_{elements}$ is unitary, due to the unitarity of $S_{elements}$ and $\Lambda(\boldsymbol{k})$, which guarantees real-valued $\varphi(\boldsymbol{k})$, as $e^{-i\varphi(\boldsymbol{k})}$ is the eigenvalue of $S(\boldsymbol{k})$. Explicitly, the formula for $S(\boldsymbol{k})$ is the matrix product:

$$S(\boldsymbol{k}) = \begin{bmatrix} 0 & e^{-i\boldsymbol{k}\cdot\boldsymbol{\alpha}_2} & 0 & & & \\ 0 & 0 & e^{-i\boldsymbol{k}\cdot\boldsymbol{\alpha}_1} & & & \\ 1 & 0 & 0 & & & \\ & & & 0 & 0 & 1 \\ & & & e^{i\boldsymbol{k}\cdot\boldsymbol{\alpha}_2} & 0 & 0 \\ & & & 0 & e^{i\boldsymbol{k}\cdot\boldsymbol{\alpha}_1} & 0 \end{bmatrix}\begin{bmatrix} & & & R & T & D \\ & & & D & R & T \\ & & & T & D & R \\ R & T & D & & & \\ D & R & T & & & \\ T & D & R & & & \end{bmatrix}, \tag{S7}$$

where $R = -1 + \frac{2}{3}\cos\xi\, e^{i\xi} + \frac{2}{3}\cos\eta\, e^{i\eta}$, $T = \frac{2}{3}(e^{-i\frac{2}{3}\pi}\cos\xi\, e^{i\xi} + e^{i\frac{2}{3}\pi}\cos\eta\, e^{i\eta})$, and $D = \frac{2}{3}(e^{i\frac{2}{3}\pi}\cos\xi\, e^{i\xi} + e^{-i\frac{2}{3}\pi}\cos\eta\, e^{i\eta})$.

II. Simulation method of arbitrary finite nonreciprocal networks

Consider an arbitrary network with N_r input/output ports and consisting of $N_{elements}$ nonreciprocal elements and links connecting them. We stress that this arbitrary network does not have to be uniform. In this part, we elaborate the simulation method for arbitrary finite nonreciprocal networks based on the scattering matrix method. We will show that this method can provide: the scattering matrix $S_{N\mathrm{r}}$ regarding these N_r ports, and the field map across the network upon known excitations at the N_r ports.

We first introduce a new building block (Fig. S1a), composed of one 3-port nonreciprocal element and three phase links. For a building block with index n, we describe its wave scattering property by a unitary scattering matrix S_n, expressed as

$$S_n|d_n\rangle = |e_n\rangle, \tag{S8}$$

where $|d_n >\equiv [d_{1n}, d_{2n}, d_{3n}]$ and $|e_n >\equiv [e_{1n}, e_{2n}, e_{3n}]$ represent wave amplitudes, defined at the three ports, in/out the building block, respectively. Therefore, there are totally $N_{elements}$ building blocks inducing $N = 3N_{elements}$ corresponding ports in the nonreciprocal network. In the following, we focus on the wave amplitude and scattering matrix regarding these N building-block ports. Following this way, the whole network is described by a unitary scattering matrix $S_N = diag[S_1, \dots, S_{N_{elements}}]$, expressed as

$$S_N|d\rangle_N = |e\rangle_N. \tag{S9}$$

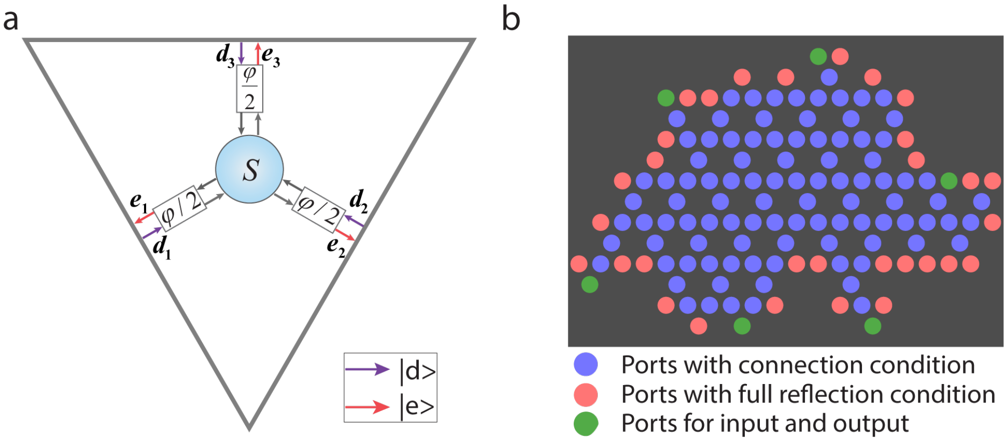

Fig. S1|Building block and port labelling used when simulating arbitrarily-shaped finite nonreciprocal networks. **a**, The individual non-reciprocal element is described by its scattering matrix S. The purple and pink arrows represent input and output wave amplitudes, denoted $|d>$ and $|e>$, respectively. **b,** We classify each inner and outer ports of the finite networks as either link, full-reflection, or input/output types, and store this information in a connectivity matrix. The panel shows an example based on the Switzerland-shaped network used in the main text.

To push forward the analysis, we label all N building-block ports as either link, full-reflection, or input/output types, as shown in Fig. S1b for the Switzerland-shaped network used in the main text. To be more specific, link-type ports are shared by two adjacent building blocks, thus with connection conditions. To extract the scattering matrix S_r for the N_r input/outer ports, at which the input and output wave amplitudes are $|d>_r$ and $|e>_r$, respectively, we label the other $N_e = N - N_r$ ports (full-reflection and link types) as eliminated ports, whose input and output wave amplitudes are still monitored, as $|d>_e$ and $|e>_e$, respectively,

$$|d\rangle_N \equiv \begin{bmatrix} |d\rangle_r \\ |d\rangle_e \end{bmatrix} \text{ and } |e\rangle_N \equiv \begin{bmatrix} |e\rangle_r \\ |e\rangle_e \end{bmatrix}. \tag{S10}$$

With the above labels, S_N can be partitioned into blocks of S_{Nr} S_{Ne}, S_{er}, and S_{re} based on the dimensions of $|d>_r$ and $|d>_e$, as

$$S_N = \begin{bmatrix} S_{Nr} & S_{er} \\ S_{re} & S_{Ne} \end{bmatrix}. \tag{S11}$$

For such N_e eliminated ports, apart from the scattering matrix in Eq. (S11), they are also subject to either connection conditions or full-reflection conditions, forming another equation:

$$S_e|d\rangle_e = |e\rangle_e. \quad \text{(S12)}$$

Combing Eq. (S9) with Eq. (S12), we get the scattering matrix S_r mapping $|d>_r$ to $|e>_r$, as

$$S_r|d\rangle_r \equiv \left[S_{Nr} + S_{er}(S_e - S_{Ne})^{-1}S_{re}\right]|d\rangle_r = |e\rangle_r. \quad \text{(S13)}$$

Having achieved the first goal of this method, namely getting the scattering matrix, we now derive the information about how wave propagate in the finite network under known excitations $|d>_{input}$ at the N_r input/outer ports. It is worth noting that Eq. (S9) and Eq. (S12) are independent, while Eq. (S13) is obtained from these two equations. We firstly combine Eq. (S12) and Eq. (S13) to an extended matrix equation involving S_A, a matrix with the same dimension than the matrix S_N, expressed as:

$$S_A = diag(S_r, S_e);\ S_A|d\rangle_N = |e\rangle_N. \quad \text{(S14)}$$

We then perform the difference between Eq. (S14) and Eq. (S9), yielding

$$\left[S_N - S_A\right]|d\rangle_N = 0. \quad \text{(S15)}$$

When a nontrival solution of $|d>_N$ is required in Eq. (S15), we need to know the Kernel[4] of $S_N - S_A$. This Kernel dimension must equal the number of input/output ports N_r, as Eq. (S14) shares the redundancy information of N_r dimensions with Eq. (S9). We then assume that a basis of the Kernel is $V = [v_1, \cdots, v_{N_r}]$, of N by N_r size. It is worthy to note that for a known excitation of the input/output ports $|d>_{input}$, the nontrivial solution $|d>_N$ must be in the Kernel. Thus, we have

$$V|f\rangle = |d\rangle_N, \quad \text{(S16)}$$

where $|f>$ is the expansion coefficient of $|d>_N$ in basis of V. In addition, as the solution $|d>_N$ contains the input amplitudes of all the ports, the input amplitudes of N_r outer ports in $|d>_N$ must

be the same with $|d>_{input}$. Following this idea, V is then partitioned into blocks of V_1 (N_r by N_r matrix) and V_2 ($N-N_r$ by N_r matrix). We can determine $|f>$ by the scattering matrix equation regarding N_r outer ports, following

$$|f\rangle = V_1^{-1}|d\rangle_{input}. \tag{S17}$$

Substituting Eq. (S17) into Eq. (S16), we finally get the wave propagation solution $|d>_N$ as

$$|d\rangle_N = VV_1^{-1}|d\rangle_{input}. \tag{S18}$$

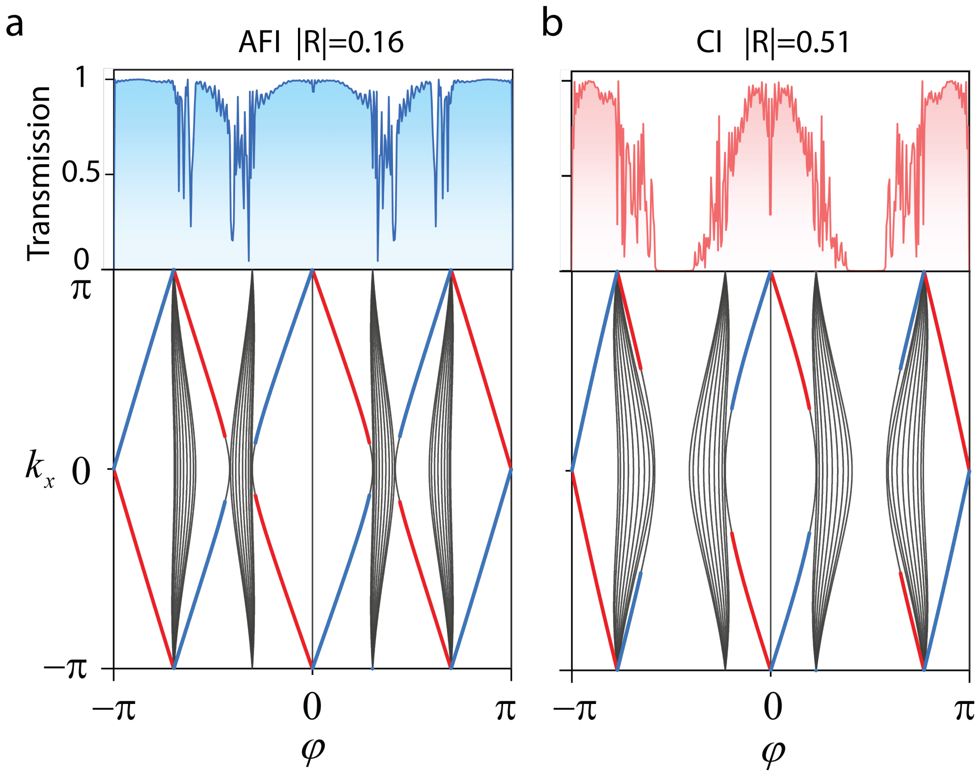

Fig. S2|Comparison between 2-port transport properties and the ribbon band structures, obtained from the semi-analytical model. We plot the transmission between Geneva and Davos through the Switzerland-shaped network as a function of φ, and compare it with the ribbon band structure. We assume a uniform distribution for the phase delay φ. **a,** case of the anomalous phase in Fig. 1c. **b,** case of the Chern phase shown in Fig. 1c. When φ falls in a topological band gap, transmission is mediated by the edge modes and reach high values. Conversely, if φ belongs to a trivial band gap, transmission is impeded. Finally, if φ falls in a bulk band, the transmission fluctuates with φ, depending on the excited bulk modal superposition interference at the output port.

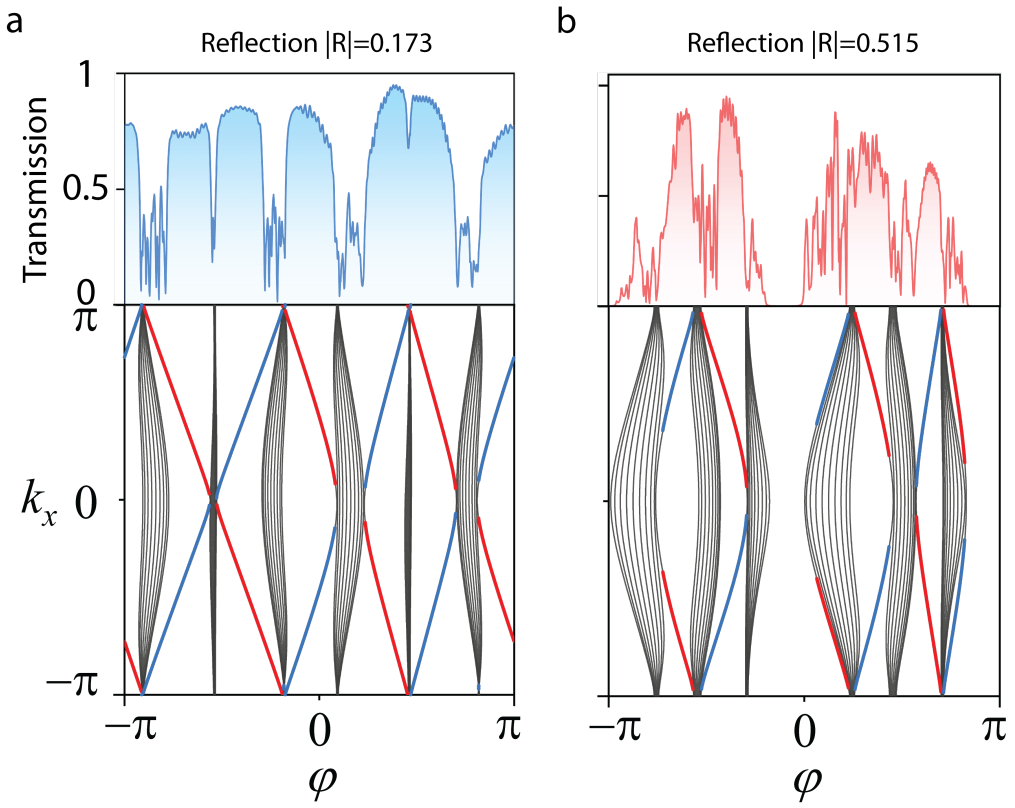

Fig. S3|Comparison between 2-port transport properties and the ribbon band structures, obtained from experimental measurements. The considered scenario is the same as Fig. S2. **a,** Case of the anomalous phase. **b,** Case of the Chern phase. The behaviour is consistent with the predictions of Fig. S2, despite small deviations that can be attributed to the slight breaking of C3 symmetry and unitarity in the experiment.

III. Examples of disorder realisations for phase-link delays and scattering matrices

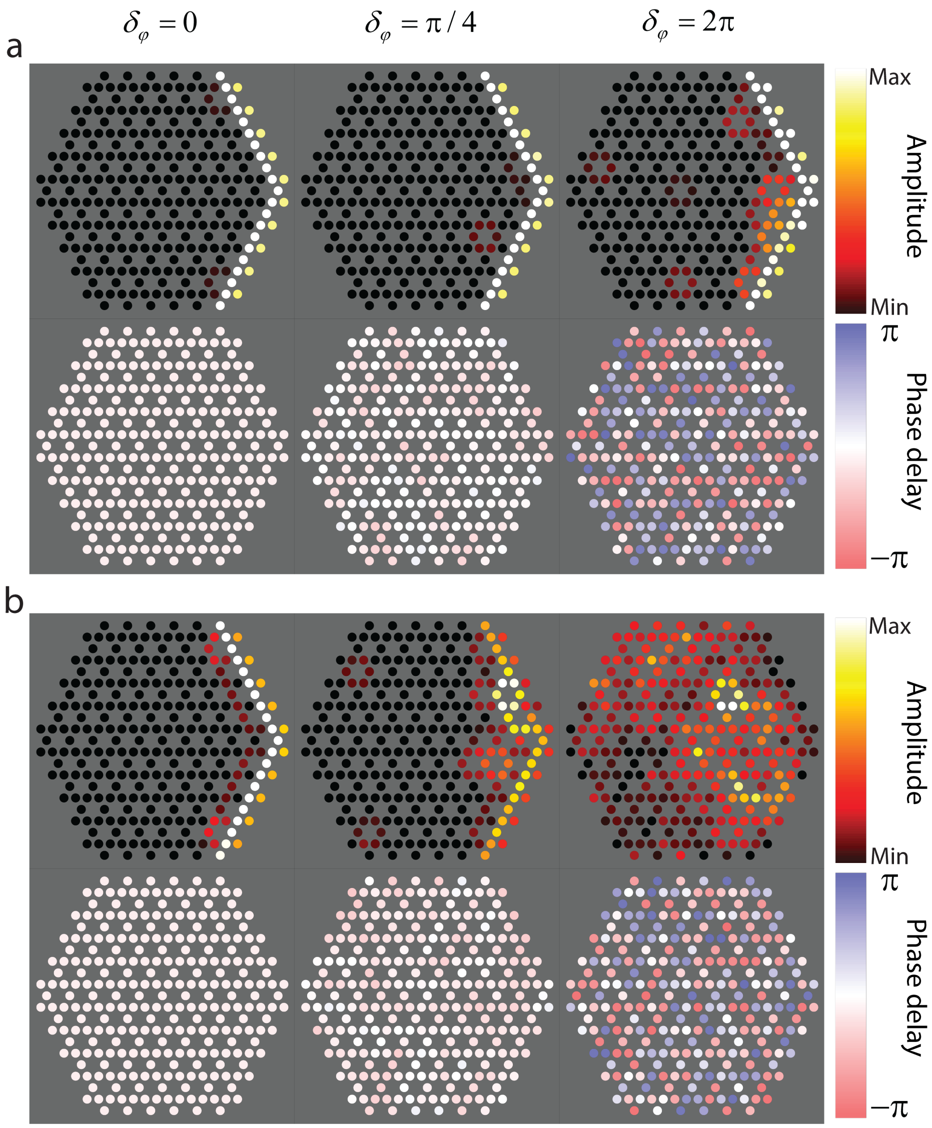

Fig. S4|Effect of phase delay disorder on the anomalous (a) and Chern (b) edge mode transmission. We consider the same hexagonal network as in Fig. 3a (left) of the main text, but now each phase link has a different delay. In both panels, the top row shows the numerically predicted field map, and the bottom row provides information about the considered particular disorder realization. The left column shows the perfectly ordered system, the middle column shows a realization of random phase delays with strength of fluctuations $\delta_\varphi = \pi/4$, and the last column is the fully disordered case, $\delta_\varphi = 2\pi$. Only the anomalous edge mode in panel a survives full random phase disorder.

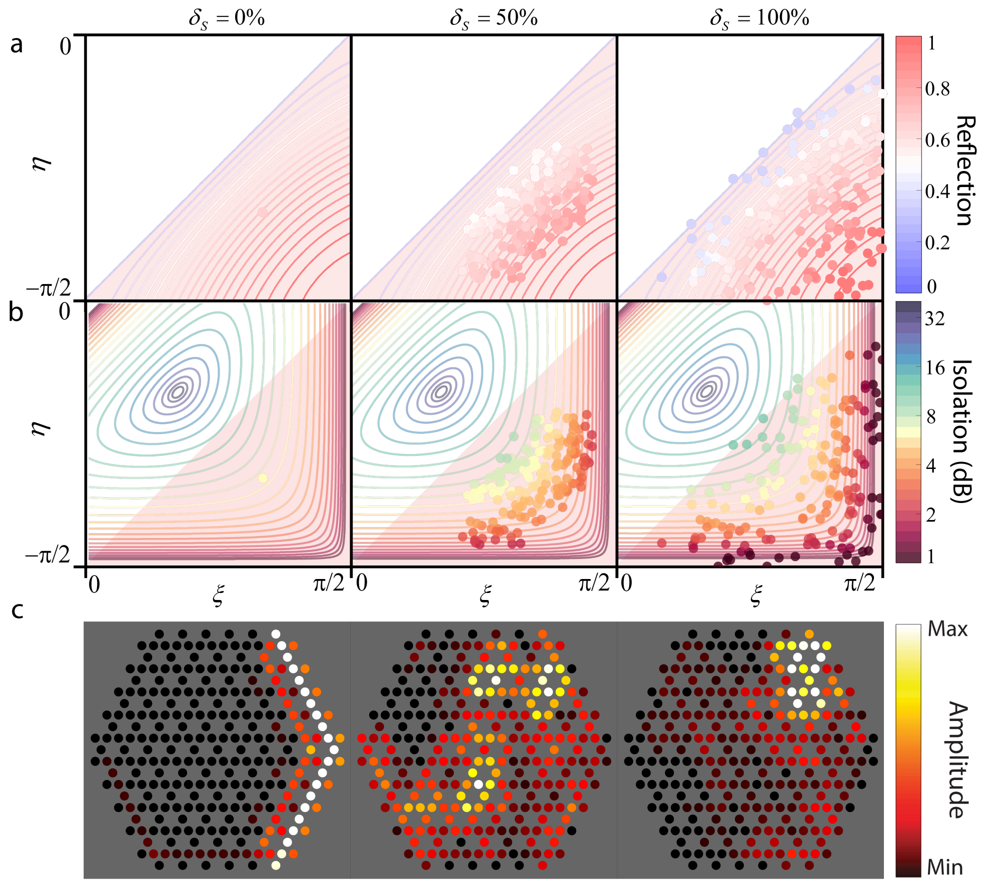

Fig. S5|Effect of scattering matrix disorder on the Chern edge mode transmission. We consider the same hexagonal network as in Fig. 3a (left) of the main text, but now each scatterer has a different scattering matrix. The left column shows the perfectly ordered system, the middle column shows a realization of random scattering disorder filling 50% of the Chern phase, and the right column shows a realization with 100% disorder, namely with scattering matrices anywhere inside the Chern phase. **a**, **b**, Repartition of the scattering matrices within the Chern phase (bottom right red triangle). The color map shows the corresponding reflection (panel a) and non-reciprocal isolation (panel b) values. **c**, Corresponding field maps, showing the sensitivity of Chern edge modes to scattering disorder.

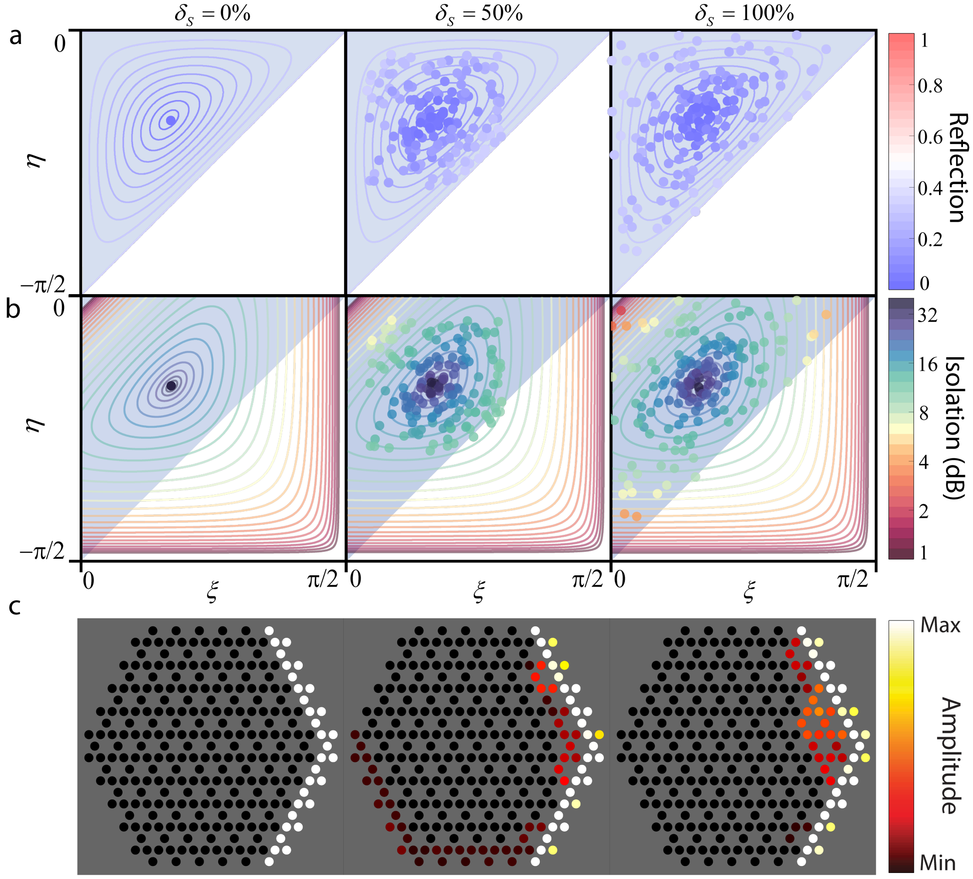

Fig. S6|Effect of scattering matrix disorder on the anomalous edge mode transmission. Same as Fig. S5 but the study is performed for the anomalous phase (top left blue triangle). Unlike the Chern one, the anomalous edge mode transmission is very robust even for fully random scattering matrix disorder.

IV. Discussion of the measured amplitudes of the scattering parameters in Extended Data Fig. 4

As shown in Extended Data Fig. 4d, between Chern-phase network 1 (N1) and network 2 (N2), there are considerable differences from 3.5 GHz to 3.9 GHz for transmission from port 1 to port 2 (S21, left), transmission from port 1 to port 3 (S31, mid), and reflection at port 2 (S22, right). These differences imply that Chern phases are not robust against strong perturbations of phase links. To be more specific, regarding N1, we see a high S21 and a low S31 denoting an edge mode from port 1 to port 2. Conversely, regarding N2, a low S21 and a high S31 imply a change of its

wave propagation path due to the perturbation of phase links in the bottom part. The high S22 (around -1 dB) of N2 indicates a trivial band gap for the bottom part of N2.

In Extended Data Fig. 4e, between anomalous-phase network 1 (N1) and network 2 (N2), in contrast to panel d, there are little differences of S parameter (S21, left; S31, mid; S22, right) between N1 and N2 under anomalous phases. It provides an additional evidence of the superior robustness of anomalous phases over Chern phases.

V. Mapping of the honeycomb lattice to an oriented graph

There exists a formal connection between a honeycomb lattice of circulators with bidirectional links and a Kagome network with oriented links. In the honeycomb circulator network, the scattering events occur at the non-ideal circulators through the scattering matrix S_0, while no backscattering occurs along the non-reciprocal links. We can equivalently consider the circulators as perfect, with the wave scattering events occurring along the links.

Such a mapping is displayed in Fig. S7 where we have highlighted the different paths of the transmitted waves with different colors (these paths are defined only for the perfect circulator case). The resulting network is a Kagome lattice with oriented links inherited from the circulators. The scattering parameters entering the $S_{elements}$ matrices are now combined to enter three 2x2 scattering matrices nodes S_1, S_2 and S_3, thus preserving the six degrees of freedoms per unit cell that yield six bands.

Importantly, this Kagome network is known to display trivial, Chern and anomalous phases[5,6]. In particular, it was shown that the existence of the anomalous phase is homotopically related to a critical symmetry point, called phase rotation symmetric point, where the bands are perfectly flat and equidistant in quasi-energy. Such remarkable points are reached when the scattering nodes S_1,

S_2 and S_3 are perfect transmitters so that the Kagome network decomposes into isolated identical loops. One of such configurations consists of isolated hexagons, that corresponds to the green points of the phase diagram in Fig. 2c for the original honeycomb lattice of circulators. This unambiguously proves that the topological phase surrounding those special points is anomalous. For completeness, we provide the band gap map of the network together with the values of the homotopy invariant W_ψ in Fig. S8.

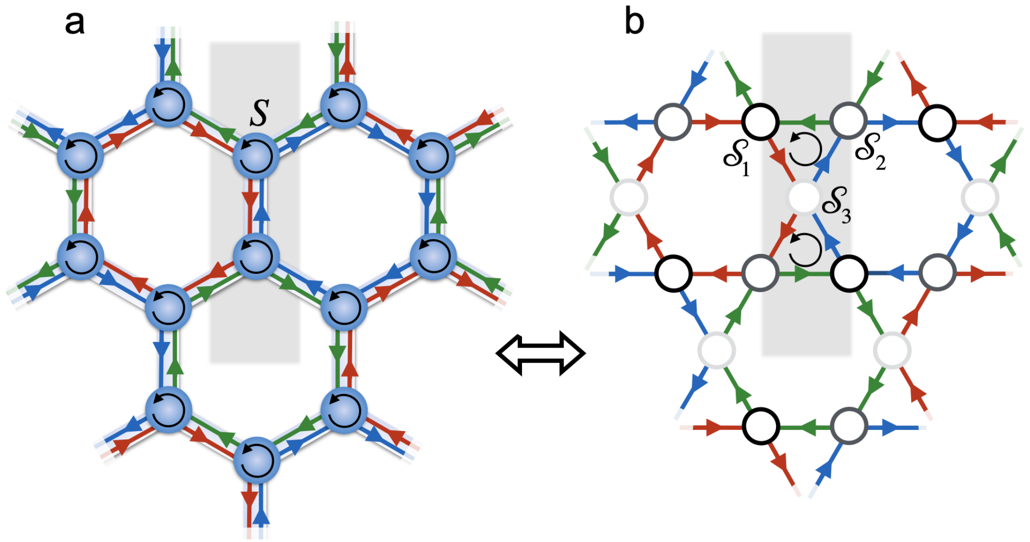

Fig. S7|Mapping of the honeycomb lattice to an oriented Kagome graph.

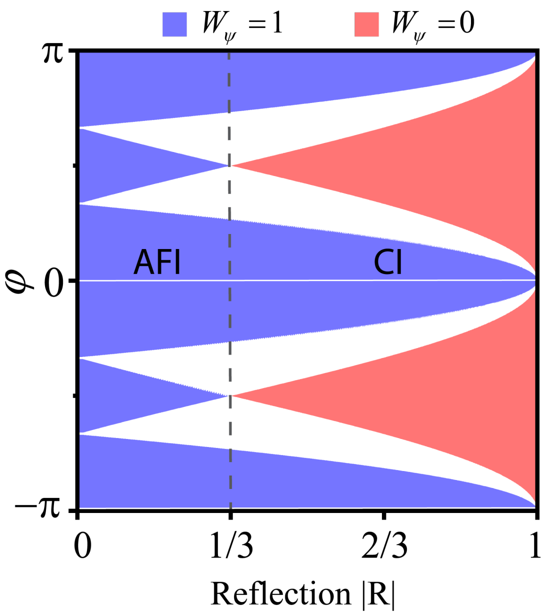

Fig. S8|Band gap map of the network. The white areas represent bulk bands. The blue areas represent band gaps with values of the homotopy invariant $W_\psi = 1$, whereas red areas correspond to band gaps with a zero value of W_ψ. R is the reflection coefficient of the individual circulators.

VI. Robustness comparisons with other Chern phases

Here, we further verify the superior transmission robustness of anomalous phase by extra comparisons with other Chern phases characterized by various types of band structures. Our extra study shows that even by working with Chern phases with less trivial band gaps or more edge states in the topological gaps, we cannot reach the same level of robustness than the anomalous phase. For easy reference, we note M the number of trivial band gaps of a band structure.

We firstly study the case of Chern phase with a single trivial band gap (M=1), which is less than the number of trivial band gaps of the Chern phase shown in the main text (M=2). In our network, such a phase cannot be directly obtained, because the matrix $S(\boldsymbol{k})$ satisfies the relation $PS(\boldsymbol{k})P^{\dagger} = -S(\boldsymbol{k})$, where $P = diag(\boldsymbol{I}_{3\times3}, -\boldsymbol{I}_{3\times3})$. The relation implies that the bulk band structures have π-translation symmetry, forcing the gaps to close and open by pairs. This π-

translation symmetry can be broken if we introduce an extra unitary 2-port reciprocal scatterer in the middle of the connecting links.

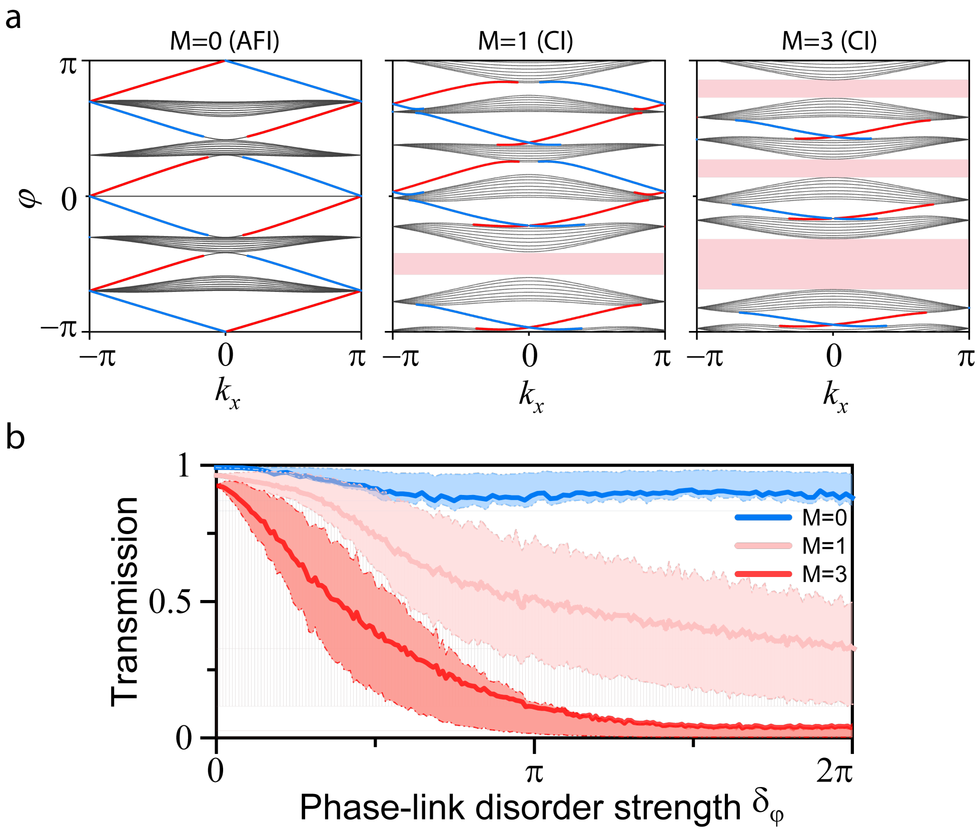

Fig. S9|Effect of the number of trivial band gaps (noted M) on the robustness of the edge mode transmission to phase link disorder. a, Ribbon band structures. We compare the case of the anomalous phase (left), in which all band gaps exhibit edge modes, to the case of a Chern phase with a single trivial gap (M=1, center), which is the most favorable scenario for Chern. For a complete comparison, we also take the case M=3 (right). The case M=2 is already studied in the main text (Fig. 3c left). **b,** Transmission statistics (average, Q1 and Q3) for the three cases for 1000 realizations of random disorder.

Specifically, by setting the reflection of this extra scatterer to 0, we recover the previous network discussed in the main text. However, playing with the reflection level of this extra scatterer allows us to extend our parameter space and reach the cases where M is even. We generate the cases M=0, 1, and 3 with their ribbon band structures shown in Fig. S9. The phase M=0 is the anomalous phase, and M=2 is the Chern phase already reported in Fig. 2a in the main

text. Next, we perform a statistical transmission analysis on a finite network with 1000 different realizations of phase link disorder, with range up to 2π. Panel b shows the results. Clearly, even the M=1 case, despite having a small trivial band gap (its size is around π/6), is largely affected by the disorder. M=3 is worse than the case M=2 presented in the main text, as expected.

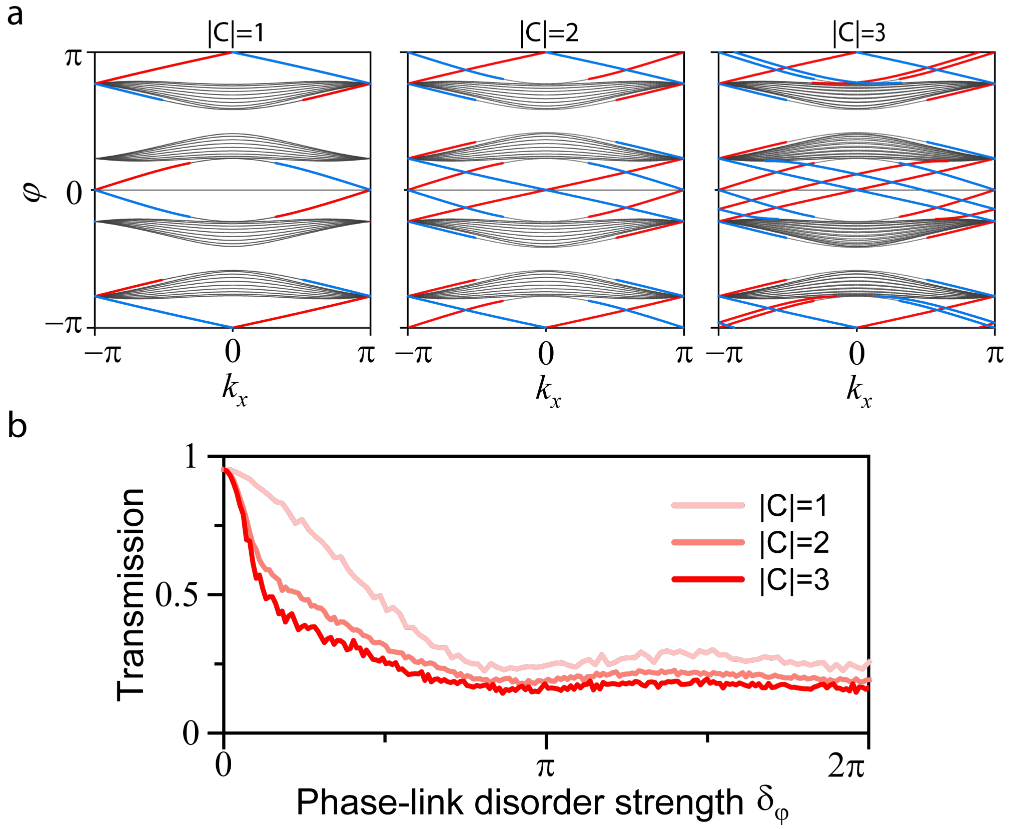

Fig. S10|Effect of the number of Chern edge modes on the transmission robustness. a, Band structures. We compare three different Chern phases with one (left), two (center) and three edge modes (right) in the non-trivial gaps. **b,** Transmission averages for 1000 realizations of random phase disorder. As for transmission between two antennas in a multimode environment, the input power has to split over the various transmission channels which interfere at the output. Disorder creates random interferences between the different channels at the output, which is statistically detrimental to transmission.

In addition, we considered the three cases shown in Fig. S10, whose band structures are characterized by Chern numbers with absolute values of 1, 2, and 3 respectively (to increase the

Chern numbers, we stacked several networks with Chern phases with $C = 1$ together and coupled adjacent layers weakly with unitary directional couplers). The Chern gaps have, consequently, 1, 2 and 3 edge modes per edge (the band structure is for a ribbon, as Fig. 2a in the main text, with edge modes at both top, in red, and bottom, in blue). Next, we consider the transmission averaged over 1000 realizations of phase link disorder for the three cases (panel b). We observe that an increase of the number of edge modes does not improve the transmission. This is actually expected: the input power has to split over the various available transmission channels. Each transmission channel will contribute to transport and undergo a different phase shift when disorder is imparted, before interfering at the output port. This interference is statistically detrimental to power transmission, a phenomenon known as multimode interference, as exploited for example in multi-mode fiber optical sensors.

Finally, we computed the average transmission for a fully-random phase-link disorder at each point of our phase diagram, i.e. for all possible tunings of the band structure (Fig. S11b). Intuitively, in the fully-random (2π strength) phase-link disorder, the value of the transmission depends on the ratio in amplitude between the bandwidths of all the bulk bands, the trivial gaps, and the gaps hosting chiral edge modes. In particular, by reducing the band widths, one increases the transmission, even for the Chern phase.

Note that this mechanism is nonetheless more efficient for the anomalous phase than for the Chern phase. Indeed, the anomalous phases are related by a continuous deformation (that does not close a gap) to a phase-rotation symmetric point, where all the bulk bands are flat[3]. Owing to the topological nature of the Chern numbers and of the W_ψ indices, the Chern phases in scattering networks cannot be continuously deformed to such a special point, otherwise the Chern numbers would be zero. Therefore, their band widths have a minimal finite value that always reduces the

transmission compared to the contribution of an edge mode. This favors the anomalous phases that can reach a perfect T=1 transmission even in the fully-random phase link disorder, by tuning the scattering parameters at the green points in Fig. S11a.

Owing to this impossibility to flatten all the bulk bands, and of course to the existence of trivial gaps, it is thus clear that the optimized Chern phase - i.e. by fine tuning the scattering parameters to maximize the topological gaps and to minimize at the same time both the trivial gaps and the band widths- cannot reach the perfect transmission T=1 in the fully-random phase-link disorder configuration.

This said, there is of course nothing that prevents a priori this optimized Chern phase to have a higher transmission than the worse fine-tuned 'anti-optimized' anomalous phase (i.e. with band widths as large as possible). The question is then: is this comparison, between the best optimized Chern phase and the worse 'anti-optimized' anomalous phase, representative of the average Chern and anomalous cases?

Fig. S11b answers this question. In Fig. S11b, we find that the anomalous phases have a typical transmission much higher than the Chern ones, and that the average transmission (over scattering parameters of the phase diagram) of the anomalous regime is also mush higher than that of the Chern phase. We also find that there are very small regions where we can pick parameters so that the transmission of the anomalous phase is smaller than the highest possible transmission of the Chern phases. However, such regions only appear close to the transition lines where the gaps are small, and both the optimized Chern and 'anti-optimized' anomalous systems collapse to a nearly insulating phase. Elsewhere, when the gaps are well resolved, the transmission in the anomalous phase is close to 1 while the transmission in the Chern phase is close to 0.

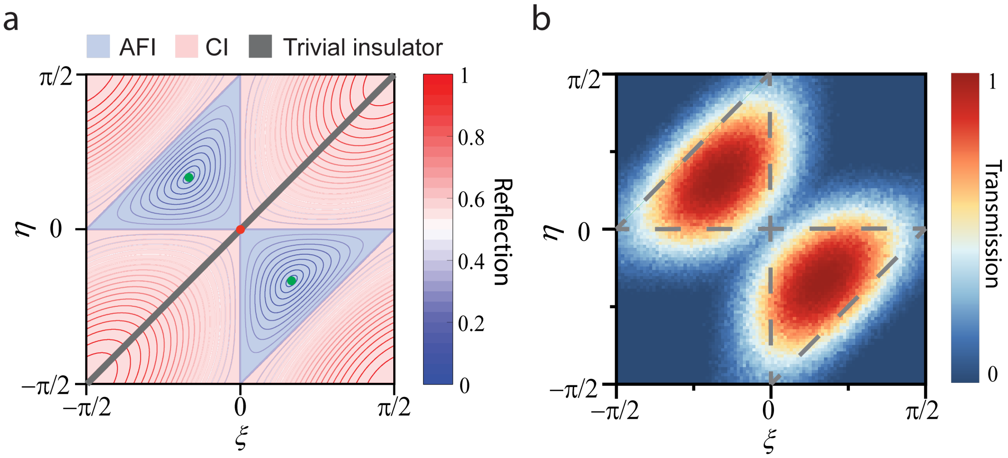

Fig. S11|Average transmission in the fully disordered phase-link case for all possible networks in the parameter space. a, Reminder of the topological phase diagram for all possible S-matrix, parametrized by ξ and η. **b**, Corresponding averaged transmission in the fully-disordered case. The average transmission in the Chern phase is much lower than the average transmission in the anomalous phase.

VII. Supplementary references

1. Nassar, H. *et al.* Nonreciprocity in acoustic and elastic materials. *Nat. Rev. Mater.* (2020) doi:10.1038/s41578-020-0206-0.
2. Fleury, R., Sounas, D. L., Sieck, C. F., Haberman, M. R. & Alu, A. Sound Isolation and Giant Linear Nonreciprocity in a Compact Acoustic Circulator. *Science* **343**, 516–519 (2014).
3. Kord, A., Sounas, D. L. & Alu, A. Microwave Nonreciprocity. *Proc. IEEE* 1–31 (2020) doi:10.1109/JPROC.2020.3006041.
4. A. Horn, R. & R. Johnson, C. *Matrix Analysis*. (Cambridge University Press, 2012).
5. Delplace, P., Fruchart, M. & Tauber, C. Phase rotation symmetry and the topology of oriented scattering networks. *Phys. Rev. B* **95**, 205413 (2017).
6. Delplace, P. Topological chiral modes in random scattering networks. *SciPost Phys.* **8**, 081 (2020).
